# Implicit processing of basic facial expressions in young children with autism: an eye-tracking study

**DOI:** 10.3389/fpsyg.2026.1815115

**Published:** 2026-05-18

**Authors:** Yu Xie, Mengyao Xin, Weina Ma

**Affiliations:** 1 Jing Hengyi School of Education, Hangzhou Normal University, Hangzhou, China; 2 Zhejiang Philosophy and Social Science Key Laboratory for Research in Early Development and Childcare, Hangzhou Normal University, Hangzhou, China

**Keywords:** autism spectrum disorder, eye-tracking, facial expressions, implicit processing, young children

## Abstract

**Background:**

Implicit processing of facial expressions by individuals serves as the foundation for accurate recognition and comprehension of emotional information. Difficulties in recognizing facial expressions was considered a typical characteristic of children with autism spectrum disorder (ASD). However, it remains unclear whether young children with autism have intact implicit processing of basic facial expressions.

**Aims:**

The present study utilized eye-tracking technology to assess the implicit processing performance of facial expressions in young children with ASD.

**Methods:**

A total of thirty young children with ASD and 30 age-matched children with typical development (TD) were recruited. All children completed an implicit emotional face task that used facial expression photographs of Chinese children.

**Results:**

No significant differences in accuracy rates between the two groups in the task. However, children with ASD showed shorter first fixation durations and lower proportion of fixation duration compared to TD children for all emotions. In addition, both groups showed a higher proportion of fixation duration and a greater number of fixations on the eye region of negative emotional faces (e.g., sad faces), as well as on the mouth region of happy faces. They showed a processing preference for the eye region of emotional faces and for fearful expressions.

**Conclusion:**

These findings indicate that children with ASD have atypical eye movements during the implicit processing of facial expressions, which may result in their difficulties in facial expression recognition.

## 1 Introduction

Autism spectrum disorder (ASD) is associated with challenges in emotion recognition (Lozier et al., 2014). Emotion recognition begins with perceiving, processing, and discriminating facial expressions (Li et al., 2024). Facial expressions are important carriers of non-verbal social information, and the processing of emotional information from faces in individuals with ASD influences both the accuracy of emotion recognition and the efficiency of understanding facial expressions, which may contribute to their difficulties in social interaction (Klara et al., 2018).

Two meta-analyses have revealed that facial emotion recognition difficulties in autistic individuals do not disappear or diminish with age; rather, they worsen as age increases (Masoomi et al., 2025; Uljarevic and Hamilton, 2013). There is substantial heterogeneity regarding which specific types of emotions are difficult to recognize, and findings vary depending on task type and variable measures (Harms et al., 2010). To the best of our knowledge, only two longitudinal studies have investigated this topic. Rosen and Lerner (2016) found that the ability to recognize basic emotional facial expressions in autistic youth aged 11–17 years improved over an 18-week period. Another study tracked the development of emotion-recognition abilities for four basic emotions in children with and without autism, aged 2.5–6 years, across 3 years (Li et al., 2024). Li et al. (2024) reported that while autistic children experienced greater difficulties in emotion recognition than their non-autistic peers, they performed similarly when required to attribute emotions based on situational cues. Furthermore, emotion recognition in the autistic group improved with age relative to their non-autistic peers. These results raise a critical question: are the emotion recognition difficulties observed in children with autism attributable to their underlying challenges in processing facial expressions?

Previous studies have mainly used explicit emotion processing tasks (e.g., emotion-matching paradigms and emotion-labeling paradigms) to investigate the processing of facial expressions in individuals with ASD. These studies contributed to our knowledge of the between-group differences. However, facial expression processing not only involves consciously explicit processing but also automatically implicit processing (Liang et al., 2022). Implicit processing of facial expression refers to the phenomenon whereby facial expressions can influence one’s attention and behavior without full conscious awareness (Yeung, 2023). In daily social interactions, the processing of others’ facial expressions often occurs unconsciously. Therefore, studying the performance of autistic children in implicitly processing of facial expressions is vital to understanding their atypical facial expression recognition.

Few studies have examined the implicit processing of facial expressions in children with autism. Currently, there are two experimental paradigms used in studying autism. First, Dawson et al. (2004) proposed the free viewing picture task, which requires participants to observe facial expressions without any response. Given the atypical attention patterns toward social stimuli (e.g., faces) in children with autism (Griffin et al., 2025), it is difficult to ensure that children with autism maintain their attention at the free viewing picture task. Second, Leung et al. (2015) proposed the implicit emotional face task, which requires participants to focus on a non-emotional target (e.g., scrambled pattern), thereby helping to maintain their attention on the task. At present, several studies have combined the two tasks with neuroimaging techniques. And these results consistently revealed that abnormal brain activation and electrophysiological responses in individuals with autism during implicitly processing of facial expressions (Batty et al., 2011; Christian et al., 2022; Dawson et al., 2004; Koelkebeck et al., 2021; Wei et al., 2025). For example, using the implicit emotional face task, Leung et al. (2019) found that, during the implicit processing of angry and happy faces, both children and adults with autism exhibited significantly lower activation in the left thalamus and right posterior cingulate cortex relative to non-autistic participants. Similarly, Kleinhans et al. (2011) used the free viewing picture task to examine brain responses in autistic adults during implicit processing of fear faces. They found that ASD group observed significantly lower activation in social brain regions (e.g., the superior colliculus, thalamus, amygdala, and fusiform gyrus) relative to the TD group (Kleinhans et al., 2011). In addition, an electroencephalography (EEG) study using a free-viewing paradigm demonstrated that TD children showed significantly larger N300 amplitudes in response to fearful faces compared to neutral faces, whereas this difference was absent in children with ASD (Dawson et al., 2004). Batty et al. (2011) used the implicit emotional face task and found that children with autism showed prolonged latencies in P1 and N170, and reduced P1 amplitudes compared to the TD group during implicit processing of basic emotional expressions (e.g., anger, disgust, happiness, sadness, surprise, and fear).

Other studies primarily used the free viewing picture task with adult emotion photographs to measure eye movement patterns during implicit processing of facial expressions in individuals with ASD. These studies have consistently reported between-group differences in implicitly processing of facial expressions, yet the specific characteristics of the atypical performance involved remain controversial. For instance, two studies have reported that children with autism aged 3 to 13 years show significantly reduced fixations and shorter fixation durations compared to the TD group (Kochhar et al., 2024; Vacas et al., 2022). Reisinger et al. (2020) utilized the same free viewing picture task and found that individuals with autism aged 3 to 21 years exhibit significantly reduced fixations and shorter fixation durations on the eye regions relative to TD controls (Reisinger et al., 2020). To our knowledge, only one study have used the implicit emotional face task (e.g., gender detection) combined with eye-tracking to investigate implicit processing of facial expressions in autism. They did not find between-group differences in fixation patterns of eye-region, but ASD group aged 10 to 18 years showed significantly increased fixation frequency on the mouth region when implicitly processing adult faces with angry, fearful, and neutral expressions (Tkalcec et al., 2025). Notably, these studies used adult facial expression photographs as experimental materials; however, research on how children with autism implicitly process peer facial expressions is lacking. On the other hand, only one study used the implicit emotional face task with adult emotion photos, and the stability of Tkalcec et al.’s results need further verification. Given the preschool period represents a critical period for the rapid maturation of facial expression processing abilities (Pons et al., 2004), it is still necessary to explore the implicitly processing performance of facial expressions in young children with autism if children’s facial expression photographs are used in the implicit emotional face task.

Accordingly, the aim of the present study was to examine the eye movement patterns of implicit processing of young children with autism and age-matched typically developing children using the implicit emotional face task with Chinese children emotion photos. In contrast to using adult faces or child faces from other ethnic backgrounds, the use of facial expression images of ethnically and age-matched Chinese children as stimulus materials in the current study yielded superior ecological validity. By analyzing the eye movement indicators (e.g., the total fixation duration, the first fixation duration, the fixation count, and the proportion of fixation duration) of two groups, we explored whether the implicit processing of facial expressions in Chinese young children with ASD was abnormal. This study tested tow hypotheses: (1) Young children with autism had abnormal implicit processing performance of facial expressions, shown as shorter fixation durations and fewer fixation counts on facial expressions. (2) Similar implicit processing performance of eye-region would be observed in both groups, but autism group would show significantly increased fixation frequency on the mouth region.

## 2 Methods

### 2.1 Participants

The sample size was calculated using GPower 3.1.9.7 (Faul et al., 2007) with an *a priori* power analysis. We set the significance level (*α*) to 0.05, statistical power (1 − *β*) to 0.80, and assumed a large effect size (Cohen’s *d* = 0.9) based on previous literature. A Cohen’s d of 0.9 aligns with prior differences between autistic and non-autistic groups, as well as between clinical and non-clinical groups in affect production and recognition (Foster et al., 2024; Kohler et al., 2008). For a repeated measures ANOVA, GPower indicated a minimum of 11 participants per group (total *N* = 22).

Thirty young Chinese children with autism (22 boys, age range: 51–96 months) were recruited from special education and rehabilitation institutions, and 30 age-matched TD children (22 boys, age range: 61–80 months) were recruited from kindergartens in Hangzhou. Given the very young age of the sample, this over-recruitment was intended to account for potential attrition and to maintain consistency with previous comparable studies (Leung et al., 2019, which included 28 autistic children and 27 TD children). The data reported in the present study were based on the final sample after excluding four autistic children (two who did not complete all experimental tasks, and two with missing eye-tracking data exceeding 5%) and one TD child, whose teacher reported hospital evaluation results indicative of ADHD. Inclusion criteria for the autistic group included an existing diagnosis of autism by a licensed psychologist or physician with extensive experience in the early identification of autistic children. All participants were free of any history of psychiatric diagnoses or acquired brain injury, had normal or corrected to-normal vision acuity and met the criterion of no prior involvement in similar experiments. The Chinese revised version of the Peabody Picture Vocabulary Test (PPVT-R), the Standard Combined Raven’s Test (CRT), and the Childhood Autism Rating Scales (CARS) were used to estimate language ability, intellectual ability and autism severity, respectively, for all participants (see Table 1). While not reaching full statistical significance in between-group differences, these marginal effects suggest lower scores in the ASD group than in the TD group on both the PPVT-R and CRT. However, significant group differences emerged in CARS scores.

**Table 1:** Demographic characteristics of participants(M(SD)).

Demographic	ASD (*n* = 30)	TD (*n* = 30)	*t*	*p*
Gender	22:8	22:8		
Age (months)	74.87(12.38)	73.13(6.04)	0.689	0.49
CARS	33.73(3.99)	17.07(2.96)	18.38^**^	0.0001
CRT Score	18.67(3.62)	20.68(4.38)	−1.93	0.06
PPVT-R Score	76.47(18.03)	83.87(14.83)	−1.75	0.08

**p < 0.01.

All the children and their parents gave written informed consent before participating. Specific data on socioeconomic status and educational attainment levels were not recorded, and community involvement was not required. All procedures performed in the study involving human participants were approved by Hangzhou Normal University Committee on Human Research Protection (HR 2022009) and were in line with the guidelines of the Declaration of Helsinki. If a child showed signs of discomfort (e.g., verbal refusal, turning away, crying) or actively refused to participate, the session was immediately paused. The experimenter offered a break or a preferred alternative activity. Children could withdraw from the study at any time without consequence.

### 2.2 Materials

The field currently lacks a validated stimulus set of emotional expressions specifically featuring Chinese children. To address this, 23 first- and second-grade typically developing children (age range 66–84 months; 11 boys) were recruited from a primary school as face models. During the photo-shooting session, models were positioned against a white background and instructed to portray four facial expressions: fear, sad, anger and happy. After the photo shoot, 92 color photographs of children’s facial expressions were obtained. Each photograph was evaluated by thirty-two undergraduate students across three dimensions: perceived facial expression type (rated on a 4-point scale: 1 = sad, 2 = fear, 3 = angry, 4 = happy), perceived emotional intensity and arousal (rated on a 9-point scale: 1 = the weakest intense/arousal, 9 = the strongest intense/arousal), and perceived affective valence (rated on a 9-point scale: 1 = most negative, 9 = most positive). Invalid photographs were screened out in accordance with the three criteria established in prior research (Liu et al., 2021): (1) the emotional intensity and arousal of photos of different emotions were similar; (2) the valence of between positive and negative emotion had significant differences; (3) the accuracy of emotion classification for fear expression images was no less than 50% and for other expression images was no less than 70%. Finally, four sets of basic facial expression photographs (equal numbers of males and females) were selected as stimuli in this study, each set contained four expressions (sad, fear, angry, happy, Table 2).

**Table 2:** The basic information of standardized emotion photographs (M(SD)).

Emotion	Number	Average intensity	Average valence	Average arousal
Angry	4	5.10 (2.25)	3.10 (1.46)	5.24 (1.89)
Happy	4	5.42 (2.49)	6.62 (1.41)	5.85 (1.76)
Sad	4	5.15 (2.36)	3.10 (1.51)	5.37 (1.83)
Fear	4	5.12 (2.53)	3.32 (1.58)	5.61 (1.92)

To create the scrambled patterns, the four sets of basic facial expression photographs mentioned above were standardized to a resolution of 520 × 700 pixels using Adobe Photoshop 25.0. Each photograph was randomly divided into 64 cells and then subjected to mosaic (15 × 15 pixels per tile) and Gaussian blur processing (with a radius of 10.0 pixels) (Klara et al., 2018). Thus, the stimuli were composed of 16 facial expression photographs and 16 scrambled patterns.

### 2.3 Procedures

The implicit emotional face task was programmed in the Experiment Builder (EB) software provided by SR Research.^1^ All stimuli were presented on a 16-inch Lenovo laptop computer, with a screen display resolution of 1,920 × 1,080 pixels. A Eyelink Portable DUO eye tracker with a sampling rate of 500 Hz was connected to the laptop to record participants’ eye movements when they completed the task. To enhance the accuracy of eye movement tracking, the tracker was configured in remote data acquisition mode, nine-point calibration and binocular tracking. If a child failed to complete the full calibration due to inattention or distress, the experimenter allowed child to take a break and then repeated the calibration attempt up to three times. Data were included only if the calibration error for each successfully registered point was less than 1.5^o^ of visual angle. Children who could not meet this criterion after three attempts were excluded from the final sample. All the participants were tested individually at their school, they were seated at 65 cm from the computer monitor.

At the beginning of this task, a photo of scrambled pattern was shown to the participants to familiarize them with the target. Participants were instructed to find the scrambled pattern and point out it as rapidly as possible. Meanwhile the experimenter pressed the corresponding keyboard button, and the display disappeared. Then, response times and accurate rate were recorded. Before commencing the implicit emotional face task, participants complete a brief practice session consisting of 6 trials (the stimuli in these trials did not appear in the formal implicit emotional face task) to familiarize them with the task. If the accurate rate reached 80% or above, they would proceed to the formal task; otherwise, the practice session was repeated.

**Figure 1:**
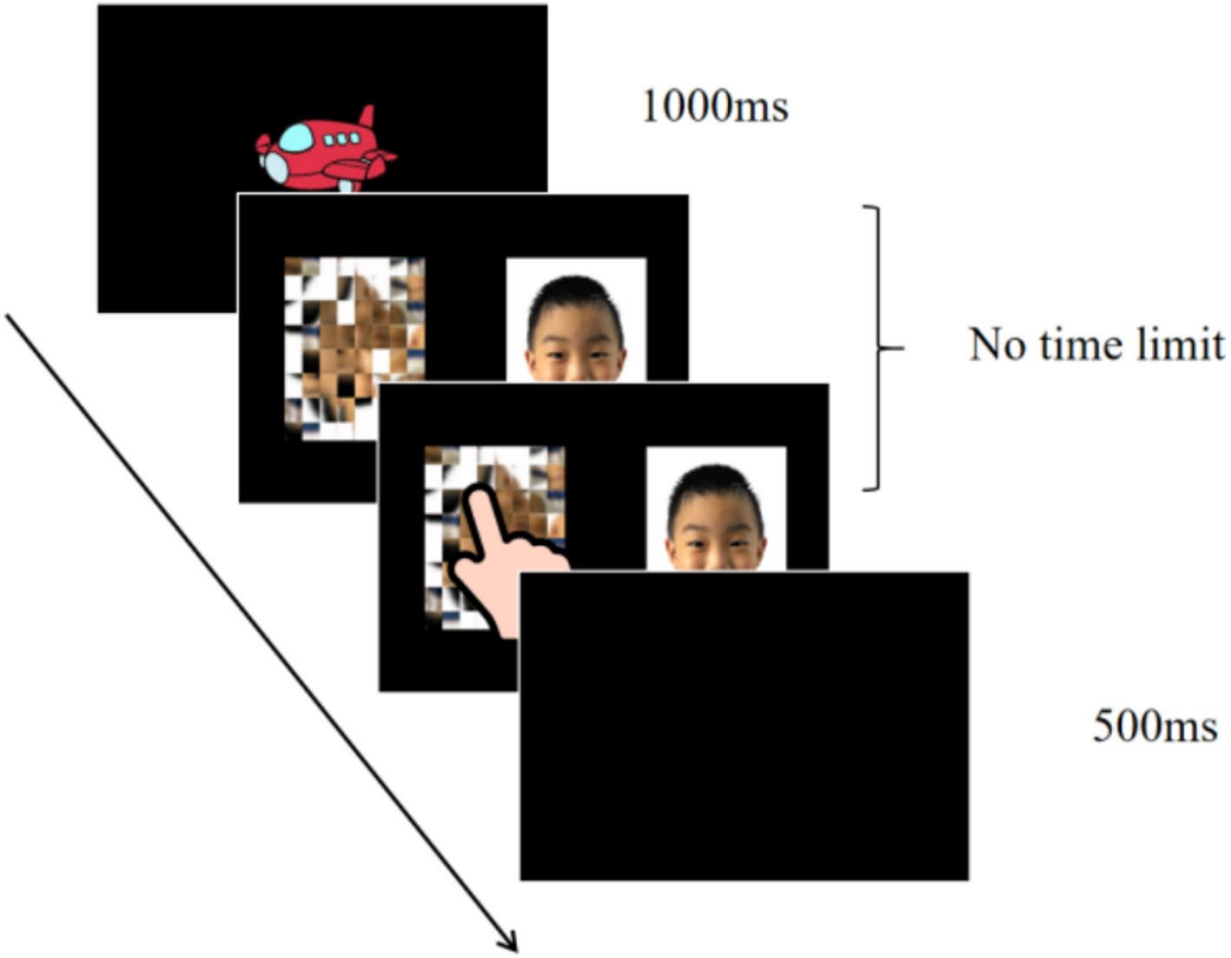
An example of trial in the implicit emotional face task.

A trial started with the presentation of a red small plane (as a fixation) cross in the middle of the screen (1,000 ms, apparent size: 7.5 × 11^o^ of visual angle), followed by two photos (one was facial expression, another was scrambled pattern). Two photos stayed on the screen until the participants point out one and the corresponding key was pressed immediately. Then, a black screen displayed 500 ms, and the next trial started (see Figure 1). A total of 32 trials were shown. The scrambled pattern (target) randomly appeared on either the left or right side, with equal probability for each location and corresponded to the “J” and “K” keys, respectively. To ensure the reliability and accuracy of data collection, a parent or teacher assisted in stabilizing the child’s head during the experiment.

### 2.4 Eye-tracking metrics

In each trial, the interval from image onset to the participant’s pointing response captured the entirety of implicit processing of the facial expression image (non-target). Accordingly, this time window was selected for the analysis of eye-tracking metrics. To measure how the participants implicitly processed facial expressions, four indicators were selected. (1) First Fixation Duration (FFD): The duration of the first fixation landing within the facial Area of Interest (AOI) during children’s implicit processing of emotional faces, which reflects the magnitude of attentional maintenance in early-stage perceptual processing; (2) Fixation Count (FC): The total number of valid fixations on the AOI during the presentation of emotional face stimuli. A fixation was defined as valid if its duration exceeded 100 ms within the AOI; The more fixation counts, the more attention children allocate to that area.; (3) Total Fixation Duration (TFD): The sum of duration of all fixations within the AOI during children’s implicit processing following the presentation of emotional faces, thus serving as an index of children’s overall attentional engagement with the AOI; and (4) Proportion of Fixation Duration (PFD): The ratio of fixation duration within a specific AOI to the total fixation duration of a single trial, which captures children’s attentional preference for facial stimuli. To avoid statistical differences in data analysis caused by inconsistent overall presentation durations of the stimulus materials, PFD was selected in reference to the analysis methods of previous studies. The specific calculation formula used in this study is as follows:

PFD = Fixation duration on a certain AOI/(Total fixation duration from facial stimulus presentation to key-press response).

### 2.5 Data preparation and analysis

All logged text data were opened using Data Viewer and then transferred to SPSS data files by the researcher. Prior to statistical analysis, the eye-tracking data were screened for completeness and quality. Following common practices in eye-tracking research, participants with more than 5% missing data for fixation measures were excluded from further analyses (Alkan, 2025). As a result, two children with autism were removed. All remaining data were inspected for outliers and normality; no additional cases were excluded.

In the study, identification accuracy and eye-tracking metrics as dependent variables was conducted. Three areas of interest (AOIs) were defined for each face stimulus (520 × 700 px) using pixel coordinates by the Data Viewer (DV) software. The Supplementary material presents the x- and y-axis boundaries for the eye, nose, and mouth regions when clear images of each emotion appear on the right side for males and females, ensuring consistent fixation mapping onto relevant facial features. For the key-press response accuracy, a correct identification of the scrambled picture by the participant was recorded as 1, and an incorrect one as 0. For the invalid trials, exclusion criteria included fixation durations exceeding ±3 standard deviations from the mean and pointing response times shorter than 500 ms (suggesting that the pointing response was too fast for a participant to have truly perceived and responded to the stimulus) (Chen et al., 2011; Fan et al., 2016). Finally, 151 trials were trimmed from the ASD group and 81 trials from the TD group, with the proportion of invalid data being approximately 0.12. All remaining data were analyzed using SPSS 25.0. To compare the ASD and TD groups in differences of eye movement patterns in the implicit processing of facial expressions, a series of repeated measures ANOVA were performed. To correct for multiple comparisons, we applied the False Discovery Rate (FDR) procedure proposed by Benjamini and Hochberg (1995) to control the expected proportion of false positives, with the FDR level set at *Q* = 0.05. Additionally, we conducted exploratory correlation analyses to examine the relationship between autism severity and eye-tracking measures.

## 3 Results

### 3.1 The accurate rate in two groups

We conducted a 4 (Emotion: sad, fear, angry and happy) × 2 (Group: ASD and TD) repeated-measures ANOVA on accurate rates (see Table 3). Results revealed that neither main effects (emotion: *F*_(3, 174)_ = 1.191, *p* = 0.315; group: *F*_(1, 58)_ = 1.552, *p* = 0.218) nor interaction between group and emotion (*F*_(3, 174)_ = 0.71, *p* = 0.547) was significant for accuracy. In addition, the mean accuracy of both groups in identifying scrambled pattern (target) exceeded 0.85, suggesting that the scrambled pattern recognition performance was similar between autistic children and their typically developing peers.

**Table 3:** Mean of accuracy in the two groups (M(SD)).

Emotion	ASD	TD
Fear	0.89 (0.07)	0.90 (0.05)
Happy	0.88 (0.08)	0.91 (0.05)
Sad	0.89 (0.09)	0.90 (0.05)
Angry	0.87 (0.09)	0.89 (0.06)

### 3.2 The eye-tracking results of implicit processing of facial expressions in two groups

We conducted a 4 (Emotion: sad, fear, angry and happy) × 2 (Group: ASD and TD) × 3 (AOIs: eye, nose and mouth) repeated-measures ANOVA on four eye-tracking metrics (TFD, FC, FFD and PFD).

**Figure 2:**
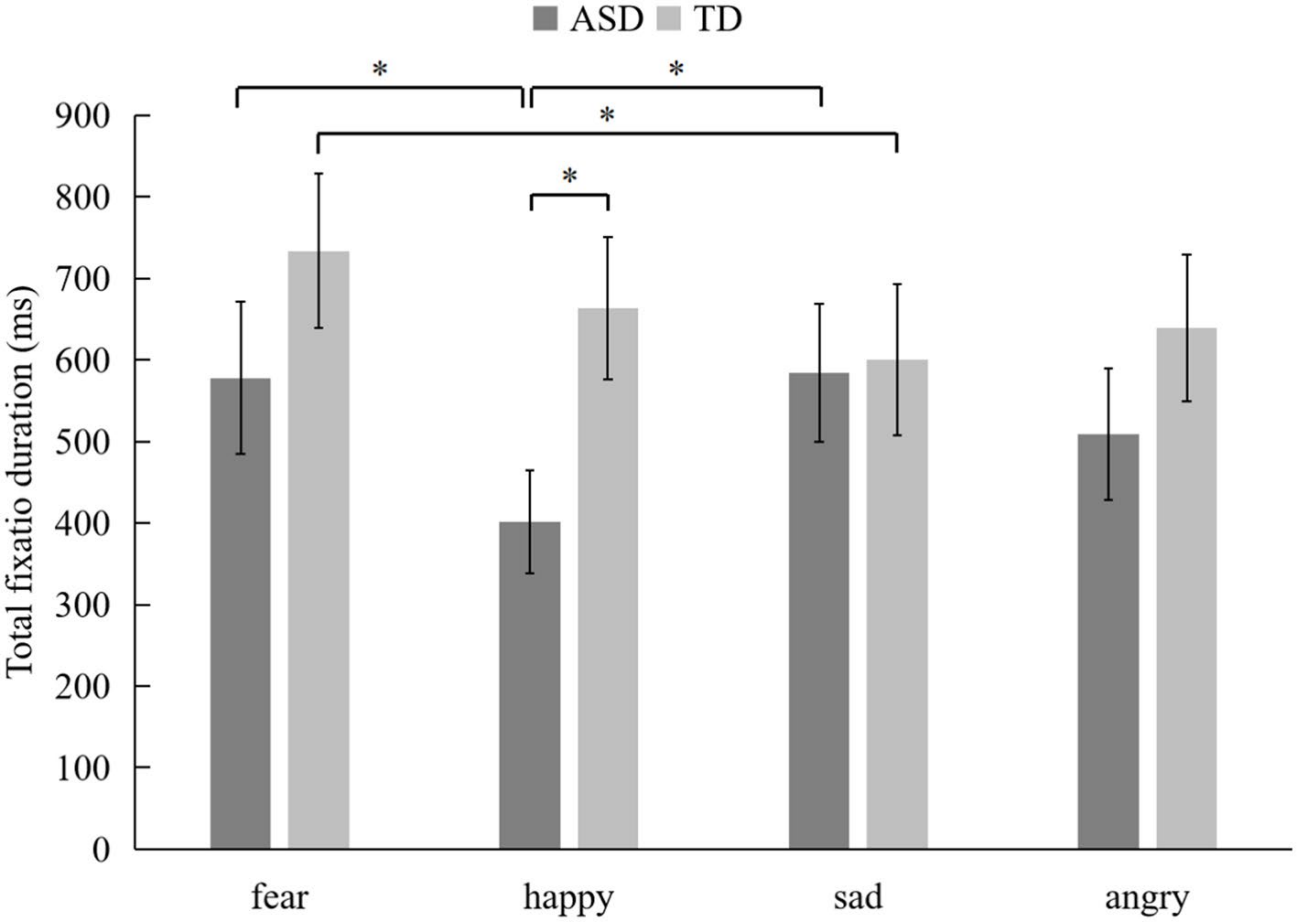
Mean total fixation duration (TFD) of implicit processing of facial expressions (*p < 0.05).

For the total fixation duration (TFD), a marginally significant interaction was found between emotion and group (*F*_(3, 522)_ = 2.479, *p* = 0.064, *η*^2^ = 0.014). Given the exploratory nature of this study, we elected to further examine this marginally significant effect by conducting follow-up post-hoc tests. Specifically, children with ASD exhibited significantly shorter TFD on the AOI of happy (*M* = 401.47, SD = 593.9) compared to both fear (*M* = 577.56, SD = 885.6, *p* = 0.013, *p*__FDR_ = 0.019) and sad (*M* = 600.09, SD = 877.04, *p* = 0.014, *p*__FDR_ = 0.021). In contrast, TD children showed significantly shorter TFD on the AOIs of sad (*M* = 600.09, SD = 806.33) relative to the AOIs of fear (*M* = 733.57, SD = 897.31, *p* = 0.024, *p*__FDR_ = 0.031). Additionally, autistic children’s TFD on the AOIs of happy were significantly shorter than those of TD children (*M* = 663.24, SD = 827.5, *p* = 0.016, *p*__FDR_ = 0.026, see Figure 2). The main effect of AOIs was also significant (*F*_(2, 174)_ = 4.522, *p* = 0.012, *p*__FDR_ = 0.017, *η*^2^ = 0.049), with both groups showing longer total fixation duration on the eye region (*M* = 795.79, SD = 1060.03) compared to the nose (*M* = 545.82, SD = 663.65, *p* = 0.049, *p*__FDR_ = 0.039) and mouth regions (*M* = 423.97, SD = 621.39, *p* = 0.004, *p*__FDR_ = 0.005). However, the main effects of emotion and group, as well as the other interaction effects, were not significant.

**Figure 3:**
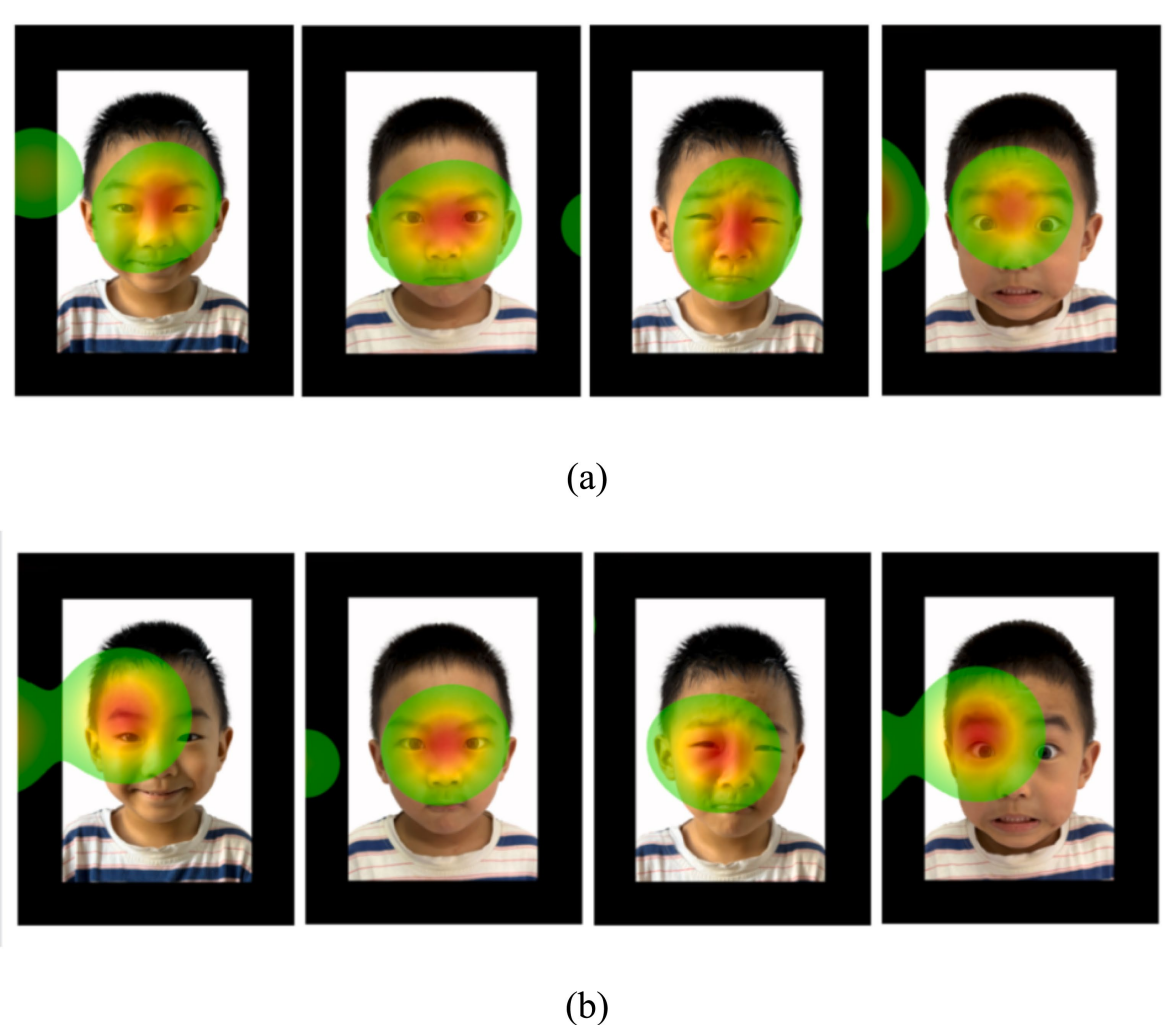
Heat maps of fixation count (FC) of implicit processing of facial expressions for a subset of TD **(a)** and ASD **(b)** groups. In these heatmaps, red and yellow areas indicate a greatest number of fixations, highest levels of interest, and most comprehensive implicit processing. Green areas represent regions with less fixations, interest and implicit processing. Blank areas show parts of the photo that had little to no fixation.

For the Fixation Count (FC), the main effect of AOIs was significant (*F*_(2, 174)_ = 6.374, *p* = 0.002, *p*__FDR_ = 0.004, *η*^2^ = 0.068). The eye region (*M* = 3.23, SD = 3.71) had higher fixation counts compared to the nose (*M* = 2.12, SD = 2.26, *p* = 0.012, *p*__FDR_ = 0.018) and mouth regions (*M* = 1.72, SD = 2.23, *p* = 0.001, *p*__FDR_ = 0.002) for both groups of children (see Figure 3).

The interaction effect between emotion types and AOI was also significant (*F*_(6, 522)_ = 2.655, *p* = 0.015, *p*__FDR_ = 0.024, *η*^2^ = 0.030). Specifically, for both groups of children, the number of fixations on the eyes of fear (*M* = 3.75, SD = 4.20), sadness (*M* = 3.33, SD = 3.54), and anger (*M* = 3.23, SD = 3.61) faces was significantly higher than that on the nose and mouth (*p*s < 0.05). The number of fixations on the mouth of happy faces (*M* = 2.067, SD = 2.39) was significantly higher than that on the mouth of sad faces (*M* = 1.4, SD = 2.10, *p* = 0.037, *p*__FDR_ = 0.036). Neither the main effects of group and emotion nor the other interaction effects reached statistical significance.

**Figure 4:**
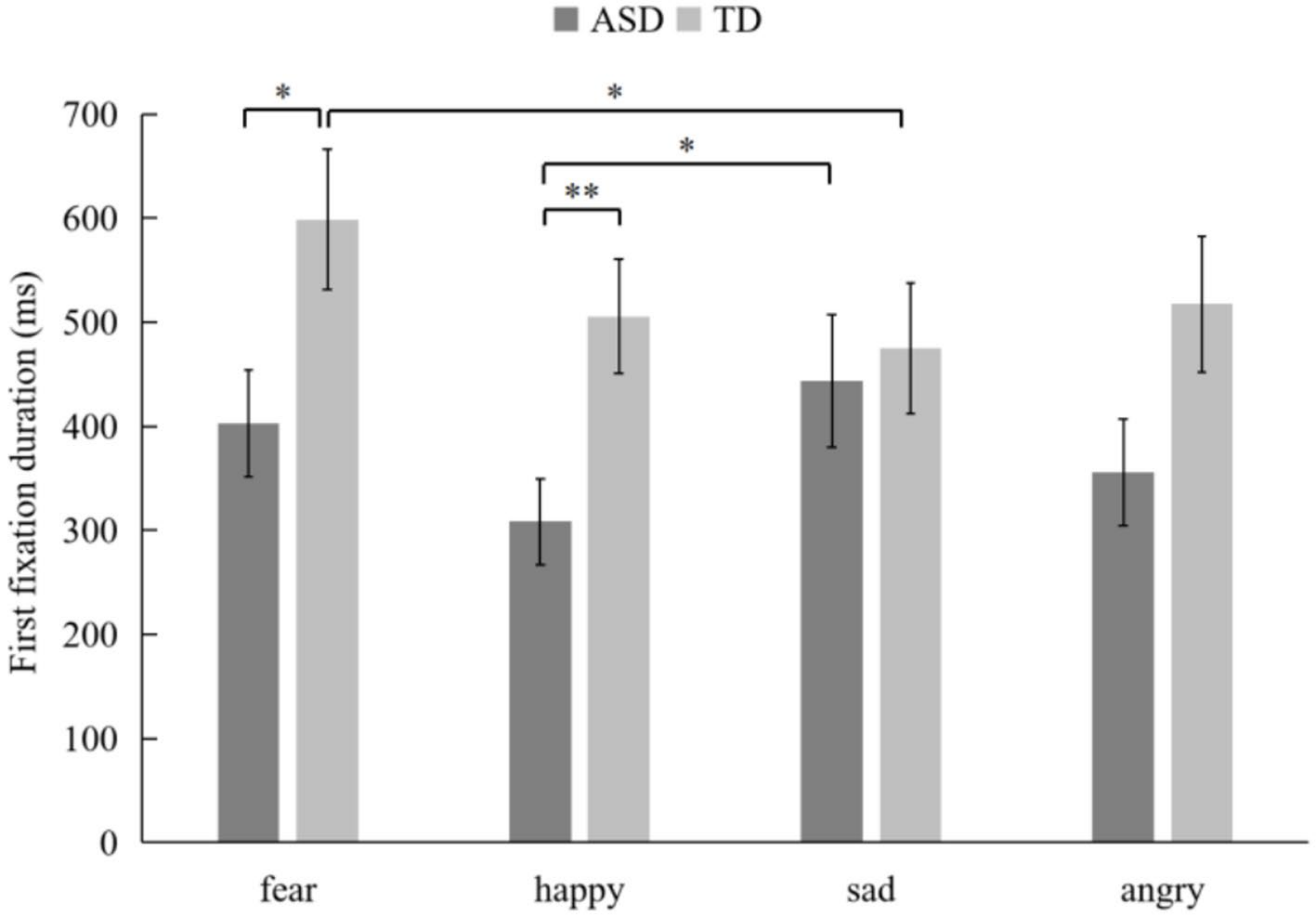
Mean first fixation duration (FFD) of implicit processing of facial expressions (*p < 0.05, **p < 0.01).

For the first fixation duration (FFD), the main effect of emotion was marginally significant (*F*_(3, 522)_ = 2.285, *p* = 0.082, *η*^2^ = 0.013). All the children exhibited a significantly longer first fixation duration on the AOIs of fear (*M* = 500.96, SD = 575.25) compared to that for happy expressions (*M* = 406.89, SD = 469.79, *p* = 0.007, *p*__FDR_ = 0.0071). The interaction between emotion and group was marginally significant (*F*_(3, 522)_ = 2.233, *p* = 0.088, *η*^2^ = 0.013). Specifically, for the ASD group, the first fixation duration on the AOIs of happy expressions (*M* = 308.26, SD = 394.29) was significantly shorter than that on the AOIs of sad expressions (*M* = 443.34, SD = 605.62, *p* = 0.021, *p*__FDR_ = 0.029). The TD children had shorter first fixation duration when they process the AOIs of sad expressions (*M* = 474.84, SD = 595.51) compared to the AOIs of fear expressions (*M* = 598.67, SD = 639.91, *p* = 0.014, *p*__FDR_ = 0.023). Moreover, as shown in Figure 4, the ASD group exhibited significantly shorter first fixation durations on the AOIs of fearful expressions (*p* = 0.023, *p*__FDR_ = 0.030) and happy expressions (*p* = 0.005, *p*__FDR_ = 0.006) than those of TD children. The main effect of group was significant (*F*_(1, 174)_ = 4.624, *p* = 0.033, *p*__FDR_ = 0.035, *η*^2^ = 0.026). Simple effects analysis indicated that, the ASD group had shorter first fixation duration (*M* = 377.72, SD = 499.37) on the AOIs of facial expressions compared to TD children (*M* = 524.11, SD = 595.36). But the main effects of AOI and the other interaction effects were not significant.

**Figure 5:**
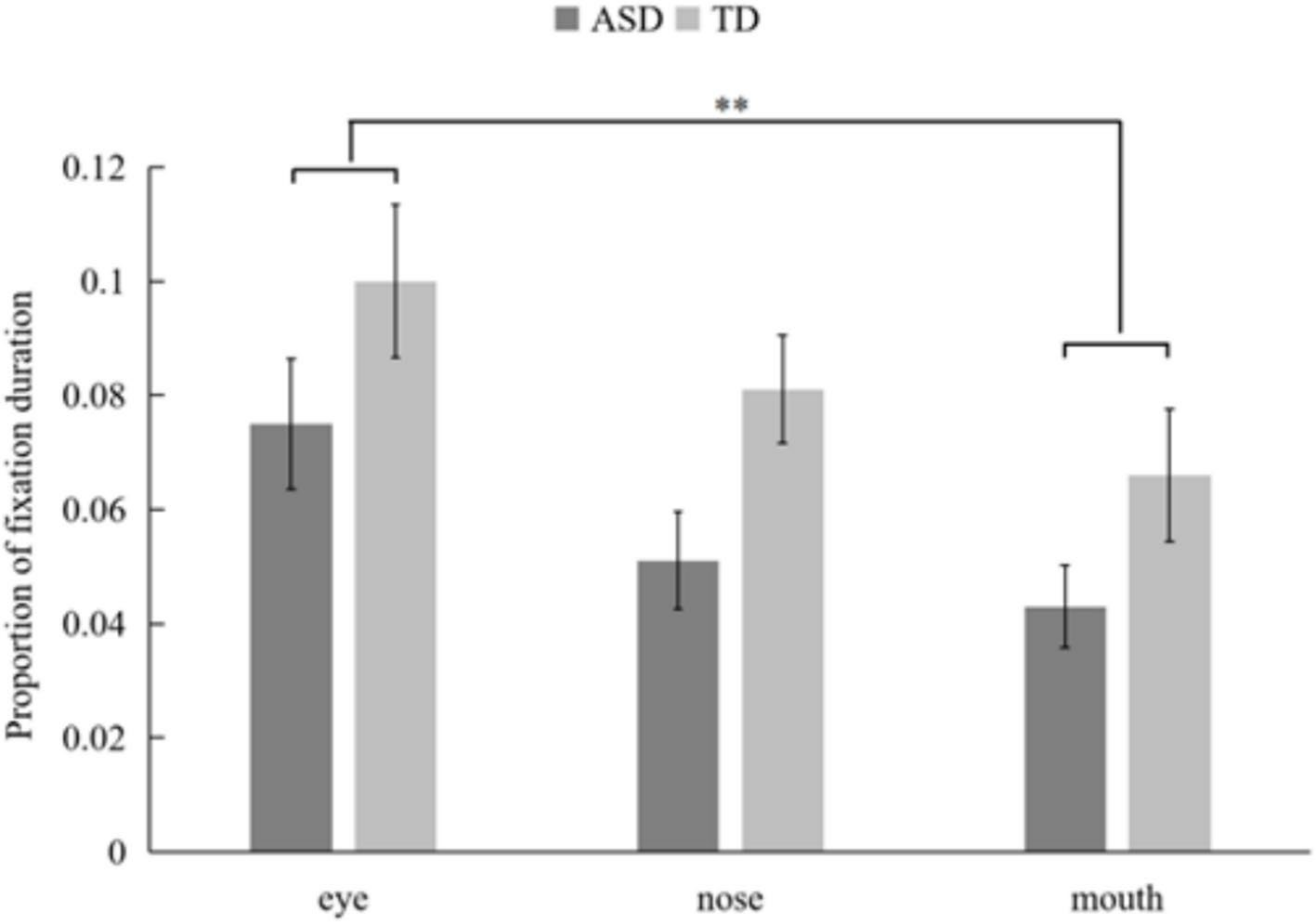
The main effect of group on the mean proportion of fixation duration (PFD) of implicit processing of facial expressions (*p < 0.05, **p < 0.01).

For the proportion of fixation duration (PFD), A significant main effect of emotion was also observed (*F*_(3, 522)_ = 3.083, *p* = 0.027, *p*__FDR_ = 0.033, *η*^2^ = 0.017), with both groups, the children showing a significantly greater PFD on the AOI for fearful expressions (*M* = 0.079, SD = 0.092) compared to that for happy expressions (*M* = 0.065, SD = 0.078, *p* = 0.008, *p*__FDR_ = 0.0083) and angry expressions (*M* = 0.064, SD = 0.079, *p* = 0.009, *p*__FDR_ = 0.011). The main effect of group was significant (*F*_(1, 174)_ = 6.785, *p* = 0.01, *p*__FDR_ = 0.012, *η*^2^ = 0.038). Simple effects analysis revealed that children with ASD (*M* = 0.056, SD = 0.072) had a significantly lower PFD on AOIs of all the emotions than TD children (*M* = 0.082, SD = 0.091). In addition, the main effect of AOI was also significant(*F*_(2, 174)_ = 3.752, *p* = 0.025, *p*__FDR_ = 0.032, *η*^2^ = 0.041). Further analysis indicated that both groups of children exhibited a significantly higher PFD on the eye region (*M* = 0.088, SD = 0.097) than on the mouth region (*M* = 0.054, SD = 0.076, *p* = 0.008, *p*__FDR_ = 0.010) (see Figure 5).

The interaction effect between emotion type and AOI also reached statistical significance (*F*_(6, 522)_ = 2.864, *p* = 0.01, *p*__FDR_ = 0.013, *η*^2^ = 0.032). Further analyses revealed that, for both groups of children, the eye region of fearful faces (*M* = 0.106, SD = 0.113) had a significantly higher PFD than both the nose region (*M* = 0.066, SD = 0.065, *p* = 0.015, *p*__FDR_ = 0.025) and the mouth region (*M* = 0.066, SD = 0.087, *p* = 0.016, *p*__FDR_ = 0.027). Additionally, the PFD for the eye region of sad faces (*M* = 0.087, SD = 0.089) and angry faces (*M* = 0.087, SD = 0.095) was significantly greater than that for the mouth region (*p*s < 0.05). The PFD for the eye region of fearful faces (*M* = 0.106, SD = 0.113) was significantly higher than that for happy faces (*M* = 0.070, SD = 0.088, *p* = 0.001, *p*__FDR_ = 0.014) and angry faces (*M* = 0.087, SD = 0.095, *p* = 0.046, *p*__FDR_ = 0.038). The PFD for the mouth region of fearful faces (*M* = 0.066, SD = 0.087) was significantly greater than that for sad faces (*M* = 0.046, SD = 0.077, *p* = 0.043, *p*__FDR_ = 0.037) and angry faces (*M* = 0.041, SD = 0.059, *p* = 0.013, *p*__FDR_ = 0.020). Furthermore, the PFD for the mouth region of happy faces (*M* = 0.065, SD = 0.078) was significantly higher than that for angry faces (*M* = 0.041, SD = 0.059, *p* = 0.01, *p*__FDR_ = 0.015). However, none of the other interaction effects were significant.

### 3.3 Correlational results

Within the ASD group, we conducted Spearman’s rank correlation analyses between four eye-tracking metrics (TFD, FFD, FC, PFD) in the eye region for four basic facial expressions and the CARS total score. Specifically, the CARS total score was negatively correlated with TFD, FFD and FC in the eye regions for fearful expression (r[30] = −0.384 ~ −0.427, all *p*s < 0.05). However, correlation analyses involving other eye-tracking data and the CARS score were not significant (see Supplementary materials).

## 4 Discussion

This study employed an implicit emotional face task combined with eye-tracking technology to investigate the implicit processing of facial expressions in young children with ASD and TD peers. The results indicated that young children with ASD exhibited shorter first fixation durations and lower proportion of fixation duration for the AOIs of four basic facial expressions compared to TD children. Meanwhile, both groups showed greater implicit processing of the eye region than the mouth region, as well as a preference for implicitly processing of fear faces over happy ones. These findings support our hypothesis that young children with ASD have atypical eye movement patterns during the implicit processing of facial expressions.

Consistent with our hypothesis, young children with ASD showed abnormalities in the implicit processing of four basic facial expressions. Specifically, compared to TD group, autistic young children had shorter first fixation durations and lower proportion of fixation duration for all AOIs across the facial expressions, alongside significantly reduced total fixation time on happy faces. This indicated that there is distinct implicit facial expression processing between groups. This result aligns with several previous studies. For instance, Vacas et al. (2022) found that young children with ASD freely fixated on adult facial expression images (happy-neutral, angry-neutral, and happy-angry pairs) for a shorter duration than TD children (Vacas et al., 2022). Similarly, Reisinger et al. (2020) reported smaller proportion of fixation duration for happy, fear, and neutral expressions in children with ASD (Reisinger et al., 2020). One influential framework for understanding these gaze patterns is the social motivation theory (Chevallier et al., 2012), which posits that abnormal facial processing in ASD arises from insufficient early social motivation, leading to reduced attention to social cues (e.g., facial expressions, body movements). However, another explanation has challenged this assumption, suggesting that behaviors such as reduced fixation duration on facial expressions may be a strategy that some autistic individuals use to focus, or to reduce or avoid stress, rather than reflecting diminished social interest per se (Jaswal and Akhtar, 2019). And reduced social attention in ASD may lead to autistic individuals spending less time attending to social stimuli (e.g., facial expressions) than typically developing controls (Chita-Tegmark, 2016).

The current study found that young children with ASD showed significantly reduced total fixation time on facial expressions, but their fixation counts were comparable to those of typically developing children. This comparable fixation counts suggests that both groups allocated a similar number of discrete attentional episodes to facial stimuli, indicating that the between-group difference in fixation duration is unlikely to be driven by a gross difference in attentional allocation. Instead, the observed reduction in fixation duration in the ASD group reflects more subtle differences in how attention is sustained over time, such as faster disengagement from or slower orientation to social cues. Goold et al. (2022) found that individuals with higher autism traits show faster disengagement of social attention during facial expression detection. Additionally, de-prioritization of social information may be an underlying mechanism driving slower looking to faces in autism (Griffin et al., 2026). Furthermore, children with autism often display a unique profile of sustained attention, characterized by intense focus on self-selected, highly interesting tasks rather than a general deficit in attention. They have difficulties in sustaining attention on imposed tasks (e.g., facial processing; Garretson et al., 1990). Therefore, these findings further confirm the uniqueness of autistic children’s eye movement patterns in implicit facial expression processing.

Notably, both autistic young children ASD and TD peers showed an implicit processing preference for the eye region. They allocated more total fixation time, exhibited higher fixation counts, and had greater proportion of fixation duration to the eye region of Chinese children’s facial expressions than to the mouth region. These findings were inconsistent with the results of previous studies (e.g., Jones et al., 2008; Klin et al., 2002; Tkalcec et al., 2025). Tkalcec et al. (2025) found that no group differences in fixation patterns of eye-region for Caucasian adults’ fear, anger, or neutral expressions, but adolescents with ASD fixated more on the mouth region than TD peers. Jones et al. (2008) and Klin et al. (2002) reported that looking at the eyes of others was significantly decreased in young children with autism. One explanation for this discrepancy is that age differences exist among the models who posed for the facial expression stimuli. Specifically, Tkalcec et al. (2025), Jones et al. (2008), and Klin et al. (2002) used facial expressions of adults, whereas our study employed facial expressions of children. It is known that individuals have more contact and interaction with peers of similar age in their daily activities. Such experiences can affect their cognitive processing in rapidly perceiving and accurately identifying others’ emotional facial expressions (Wei et al., 2023). Previous eye-tracking studies have reported that children with autism gaze longer at own-age faces than at other-age faces, and they have an attentional bias toward own-age faces (Yu, 2019). Hauschild et al. (2020) investigated whether a facial emotion recognition task would elicit an own-age bias for adolescents with and without autism. Their results indicated that an own-age bias was evident in all adolescents, regardless of ASD status or symptoms, suggesting that face processing abilities of adolescents with autism may be influenced by experience with specific categories of stimuli. In addition, Jones et al. (2008) and Klin et al. (2002) used dynamic videos in which children with autism could both see the actors’ facial expressions and hear the corresponding audio. The presence of auditory input may have drawn the children’s attention to the actors’ mouth movements, potentially leading to reduced fixation on the eye region. On the other hand, adolescents with autism may gaze more at the mouth region of adult faces as a compensatory strategy to alleviate anxiety associated with reduced eye contact (Dirk et al., 2006).

Furthermore, a higher proportion of fixation duration and a greater number of fixations on the eye region of negative emotional faces (e.g., sad faces), as well as on the mouth region of happy faces, were shown in the two groups of participants. This finding was consistent with that of Lin et al. (2022), who recorded the fixation duration of children with ASD when viewing facial images in dynamic contexts. They found that both children with ASD and typically developing children fixated significantly longer on the eye region of sad faces than on the mouth region or on the eye region of happy expressions. Thus, both groups relied more heavily on the eye region when processing negative facial expressions, reflecting that autistic children have similar implicit processing characteristics to their peers. This pattern aligns with the notion that the mouth was the most important facial area for positive emotions (e.g., happy), while the eyes were better at conveying negative emotions like fear and anger (Li et al., 2017). Enhanced implicit processing of the eye region in both children with ASD and TD children facilitates efficient capture of threatening emotional information from human faces.

Additionally, both groups demonstrated a processing preference toward threatening facial expressions in the implicit emotional face task. For total fixation time, young children with ASD spent more time processing the fear and sad expressions than happy expressions, while TD children fixated longer on fear expressions than sad expressions. Moreover, both groups also showed longer first fixation durations and higher proportion of fixation duration for fear expressions than happy ones. Similarly, Wagner et al. (2020) found that high-risk autistic infants spent more time looking at fear expressions than at the neutral and happy expressions of children (Wagner et al., 2020). Vacas et al. (2022) found that compared with happy and neutral expressions, angry expressions attract more visual attention from both young children with ASD and TD group (Vacas et al., 2022). Also, another study reported that high-function autistic children fixated longer on disgust faces, with comparable fixation times for happy and neutral faces (Hayley et al., 2015). These findings support the theory of biological evolution, which indicate that compared to other emotional expressions, threatening expressions (e.g., fear, anger, and disgust) are more likely to draw the attention and processing of individuals. This special implicit processing preference is crucial for facilitating an individual’s adaptation to social survival and maintenance of social participation (Fan et al., 2020).

Previous research has found that symptom severity in individuals with ASD moderates their face processing patterns (Sasson et al., 2008; Jones et al., 2008). For example, Sasson et al. (2008) reported that composite repetitive behavior scores were negatively correlated with perseveration on social images. Jones et al. (2008) used Autsim Diagnostic Observation Schedule (ADOS) to measure autism severity in their ASD group and revealed that fixation on eyes by the children with autism was correlated with their level of social disability. Similarly, the present study found that the Childhood Autism Rating Scale (CARS) total score was negatively correlated with TFD, FFD and FC in the eye regions for fearful expression. Therefore, children with greater autism symptom severity tend to exhibit reduced total fixation duration, shorter first fixation duration, and fewer fixation counts when viewing fearful facial expressions.

## 5 Limitations and future work

The current study has some limitations. First, the gender of participants did not match. The significantly higher prevalence of autism in males than in females, with an estimate ratio of 4:1 (Zhou et al., 2020), consequently led to a higher proportion of males in the ASD group recruited in this study. Similarly, the control group also had an uneven gender distribution. Research has found that girls with ASD may have more severe impairments in processing social information than boys with ASD (Coffman et al., 2015). Therefore, whether there are gender differences in the implicit processing of basic facial expressions in young children with autism remains to be further explored. Second, the types of emotions in the experimental materials were limited. This study only examined the four basic emotions of happy, sad, anger, and fear. Due to the young age of the participants, more complex facial emotions (e.g., guilt and pride) and body emotions were not included. In natural social interactions, individuals typically rely on both complex facial expressions and body movements to convey information. Therefore, future studies can combine complex emotional faces and body emotions materials to set up more natural and valid experimental environment, thereby further exploring the implicit processing characteristics of children with ASD for diverse types of emotional information. Third, the present study did not examine first fixation latency to the areas of interest. This metric would have provided valuable information about the speed of initial attention orienting to different facial features. Fourth, the implicit emotional face processing paradigm used in this study was originally developed by Leung et al. (2015), in which preschool children with autism were instructed to point to the target (a scrambled pattern) as quickly as possible. In this task, participants processed concurrently presented facial expressions without any explicit instruction, meaning that facial expression processing occurred incidentally. Therefore, the present experimental task did not adequately distinguish between implicit and incidental processing. Finally, it remains uncertain whether Chinese preschool children with ASD differ in their implicit processing of facial expressions of peers from other racial backgrounds. Previous studies using adult facial expression stimuli have demonstrated that, unlike their typically developing peers, children with ASD do not display an own-race advantage (Chien et al., 2014; Yi et al., 2016). Accordingly, future research is necessary to examine whether own-race effects emerge in the implicit processing of child facial expressions.

## 6 Conclusions and implications

Combining the implicit emotional face task with eye-tracking technology, this study helps reveal how children with ASD implicitly process facial expressions, while also verifying the importance of eye-tracking technology in assessing the facial expression processing ability of young children with autism. Young children with ASD exhibited atypical implicit processing of facial expressions, as evidenced by shorter first fixation durations and a lower proportion of fixation duration compared to TD children, and this pattern was observed across all emotions. However, there were similar characteristics in some aspects, namely, both groups demonstrated a processing preference for the eye region of facial expressions as well as for fear expressions. These findings contribute to revealing the causes of difficult facial expression recognition and provide empirical support for early screening in children with ASD.

## Data Availability

The raw data supporting the conclusions of this article will be made available by the authors, without undue reservation.

## Footnotes

1
https://www.sr-research.com/experiment-builder/
