# Supplementary material for: Implicit processing of basic facial expressions in young children with autism: an eye-tracking study
Source: Front Psychol. 2026 May 18;17:1815115. doi: 10.3389/fpsyg.2026.1815115 (PMC13223034; doi:10.3389/fpsyg.2026.1815115)
Supplement: Supplementary file 1 [file Table_1.docx]

TABLE 1 AOI Coordinates (in pixels) for eyes, nose, and mouth region stimulus images (image size=520 x700 px)

| Emotion | Stimulus Images | Eyes (x)  [x_1_, x_2_] | Eyes (y)  [y_1_, y_2_] | Nose (x)  [x_1_, x_2_] | Nose (y)  [y_1_, y_2_] | Mouth (x)  [x_1_, x_2_] | Mouth (y)  [y_1_, y_2_] |
| --- | --- | --- | --- | --- | --- | --- | --- |
| Happy | Male 1 | 1294, 1607 | 440, 565 | 1395, 1509 | 573, 655 | 1349, 1552 | 663, 725 |
|  | Male 2 | 1283, 1590 | 481, 576 | 1381, 1487 | 578, 663 | 1364, 1515 | 674, 747 |
|  | Female1 | 1293, 1590 | 464, 573 | 1383, 1498 | 573, 659 | 1359, 1526 | 672, 738 |
|  | Female2 | 1285, 1592 | 473, 589 | 1375, 1502 | 591, 661 | 1348, 1537 | 666, 738 |
| Anger | Male 1 | 1278, 1612 | 462, 598 | 1392, 1509 | 604, 688 | 1379, 1524 | 697, 769 |
|  | Male 2 | 1300, 1594 | 508, 600 | 1394, 1493 | 602, 681 | 1375, 1509 | 686,753 |
|  | Female1 | 1274, 1583 | 534, 642 | 1366, 1480 | 648, 732 | 1357, 1493 | 742, 800 |
|  | Female2 | 1278, 1583 | 510, 628 | 1373, 1487 | 629, 701 | 1348, 1518 | 703, 775 |
| Sad | Male 1 | 1307, 1618 | 472, 593 | 1397, 1522 | 593, 690 | 1386, 1522 | 708, 764 |
|  | Male 2 | 1272, 1583 | 494, 615 | 1362, 1474 | 618, 696 | 1351, 1506 | 707, 776 |
|  | Female1 | 1272, 1583 | 481, 596 | 1368, 1485 | 593, 688 | 1351, 1504 | 696, 776 |
|  | Female2 | 1274, 1590 | 503, 620 | 1375, 1489 | 622, 694 | 1359, 1507 | 703, 784 |
| Fear | Male 1 | 1254, 1607 | 470, 620 | 1364, 1487 | 622, 723 | 1351, 1520 | 758, 826 |
|  | Male 2 | 1283, 1605 | 464, 607 | 1375, 1502 | 609, 690 | 1361, 1529 | 701, 791 |
|  | Female1 | 1283, 1596 | 475, 609 | 1379, 1493 | 609, 692 | 1370, 1507 | 708, 802 |
|  | Female2 | 1287, 1588 | 438, 589 | 1383, 1493 | 591, 672 | 1375, 1515 | 681, 795 |

Note: All the AOIs were rectangle, x_1_=x_min, x_2_=x_max, y_1_=y_min, y_2_=y_max
