# Supplementary material for: Implicit processing of basic facial expressions in young children with autism: an eye-tracking study
Source: Front Psychol. 2026 May 18;17:1815115. doi: 10.3389/fpsyg.2026.1815115 (PMC13223034; doi:10.3389/fpsyg.2026.1815115)
Supplement: Supplementary file 2 [file Table_2.docx]

**TABLE 1. Correlation results between symptom severity in children with ASD and TFD, FFD, FC, and PFD in the eye region across four basic facial expressions.**

|  | **fear_**  **TFD** | **fear_**  **FFD** | **fear_**  **FC** | **fear_**  **PFD** | **happy_**  **TFD** | **happy_**  **FFD** | **happy_**  **FC** | **happy**  **_PFD** | **sad_**  **TFD** | **sad_**  **FFD** | **sad_**  **FC** | **sad_**  **PFD** | **angry_**  **TFD** | **angry_**  **FFD** | **angry_**  **FC** | **angry_**  **PFD** |
| --- | --- | --- | --- | --- | --- | --- | --- | --- | --- | --- | --- | --- | --- | --- | --- | --- |
| **CARS** | -.384*  (*p*=0.036) | -.388*  *(p*=0.034) | -.427*  (*p*=0.019) | -0.317  (*p*=0.088) | -0.232  (*p*=0.218) | -0.239  (*p*=0.203) | -0.186  (*p*=0.325) | -0.17  (*p*=0.369) | -0.003  (*p*=0.987) | -0.086  (*p*=0.65) | -0.082  (*p*=0.667) | -0.007  (*p*=0.972) | -0.088  (*p*=0.644) | 0.031  (*p*=0.869) | -0.210  (*p*=0.265) | -0.114  (*p*=0.549) |

注:**p*<0.05

**TABLE 2. Correlation results between symptom severity in children with ASD and TFD, FFD, FC, and PFD in the nose region across four basic facial expressions.**

|  | fear_  TFD | fear_  FFD | fear_  FC | fear_  PFD | happy_  TFD | happy_  FFD | happy_  FC | happy_  PFD | sad_  TFD | sad_  FFD | sad_  FC | sad_  PFD | angry_  TFD | angry_  FFD | angry_  FC | angry_  PFD |
| --- | --- | --- | --- | --- | --- | --- | --- | --- | --- | --- | --- | --- | --- | --- | --- | --- |
| CARS | -0.084  (*p*=0.660) | -0.075  (*p*=0.694) | -0.040  (*p*=0.832) | -0.042  (*p*=0.825) | 0.052  (*p*=0.785) | 0.003  (*p*=0.989) | 0.083  (*p*=0.661) | 0.036  (*p*=0.851) | -0.030  (*p*=0.875) | -0.071  (*p*=0.708) | 0.006  (*p*=0.973) | -0.032  (*p*=0.865) | -0.123  (*p*=0.519) | -0.103  (*p*=0.589) | -0.218  (*p*=0.247) | -0.121  (*p*=0.523) |

**TABLE 3. Correlation results between symptom severity in children with ASD and TFD, FFD, FC, and PFD in the mouth region across four basic facial expressions.**

|  | **fear_**  **TFD** | **fear_**  **FFD** | **fear_**  **FC** | **fear_**  **PFD** | **happy_**  **TFD** | **happy_**  **FFD** | **happy_**  **FC** | **happy_**  **PFD** | **sad_**  **TFD** | **sad_**  **FFD** | **sad_**  **FC** | **sad_**  **PFD** | **angry_**  **TFD** | **angry_**  **FFD** | **angry_**  **FC** | **angry_**  **PFD** |
| --- | --- | --- | --- | --- | --- | --- | --- | --- | --- | --- | --- | --- | --- | --- | --- | --- |
| **CARS** | 0.132  (*p*=0.485) | 0.203  (*p*=0.283) | 0.209  (*p*=0.268) | 0.087  (*p*=0.647) | 0.113  (*p*=0.552) | 0.123  (*p*=0.518) | 0.093  (*p*=0.626) | -0.028  (*p*=0.885) | 0.087  (*p*=0.647) | 0.102  (*p*=0.592) | 0.082  (*p*=0.668) | 0.071  (*p*=0.71) | -0.068  (*p*=0.721) | -0.126  (*p*=0.508) | -0.136  (*p*=0.473) | -0.071  (*p*=0.710) |

**TABLE 4. Correlation results between symptom severity in children with ASD and TFD, FFD, FC, and PFD for four basic facial expressions.**

|  | **fear_**  **TFD** | **fear_**  **FFD** | **fear_**  **FC** | **fear_**  **PFD** | **happy_**  **TFD** | **happy_**  **FFD** | **happy_**  **FC** | **happy_**  **PFD** | **sad_**  **TFD** | **sad_**  **FFD** | **sad_**  **FC** | **sad_**  **PFD** | **angry_**  **TFD** | **angry_**  **FFD** | **angry_**  **FC** | **angry_**  **PFD** |
| --- | --- | --- | --- | --- | --- | --- | --- | --- | --- | --- | --- | --- | --- | --- | --- | --- |
| **CARS** | -0.284  (*p*=0.128) | -0.173  (*p*=0.361) | -0.270  (*p*=0.149) | -0.226  (*p*=0.229) | -0.098  (*p*=0.608) | -0.107  (*p*=0.575) | -0.026  (*p*=0.893) | -0.128  (*p*=0.501) | 0.019  (*p*=0.922) | -0.045  (*p*=0.812) | 0.051  (*p*=0.788) | 0.042  (*p*=0.827) | -0.137  (*p*=0.470) | -0.093  (*p*=0.625) | -0.280  (*p*=0.134) | -0.177  (*p*=0.348) |

**TABLE 5. Correlation results between symptom severity in children with ASD and TFD, FFD, FC, and PFD across three regions of interest.**

|  | **eye_TFD** | **eye_FFD** | **eye_FC** | **eye_PFD** | **nose_TFD** | **nose_FFD** | **nose_FC** | **nose_PFD** | **mouth_TFD** | **mouth_FFD** | **mouth_FC** | **mouth_PFD** |
| --- | --- | --- | --- | --- | --- | --- | --- | --- | --- | --- | --- | --- |
| **CARS** | -0.192  (*p*=0.309) | -0.194  (*p*=0.305) | -0.278  (*p*=0.137) | -0.196  (*p*=0.298) | -0.175  (*p*=0.354) | -0.173  (*p*=0.36) | -0.099  (*p*=0.603) | -0.083  (*p*=0.664) | 0.037  (*p*=0.844) | 0.076  (*p*=0.689) | -0.003  (*p*=0.986) | -0.044  (*p*=0.816) |

**TABLE 6. Correlation results between symptom severity in children with ASD and TFD, FFD, FC, and PFD for facial expressions.**

|  | **TFD** | **FFD** | **FC** | **PFD** |
| --- | --- | --- | --- | --- |
| **CARS** | -0.115(*p*=0.543) | -0.109(*p*=0.566) | -0.165(*p*=0.384) | -0.134(*p*=0.482) |
